# Interspecific gamete compatibility and hybrid larval fitness in reef-building corals: Implications for coral reef restoration

**DOI:** 10.1038/s41598-019-41190-5

**Published:** 2019-03-18

**Authors:** Wing Yan Chan, Lesa M. Peplow, Madeleine J. H. van Oppen

**Affiliations:** 1 Australian Institute of Marine Science, Townsville MC, QLD, 4810 Australia; 2 School of BioSciences, University of Melbourne, VIC, 3010 Australia

## Abstract

Climate warming is a major cause of the global decline of coral reefs. Active reef restoration, although still in its infancy, is one of several possible ways to help restore coral cover and reef ecosystem function. The deployment of mature coral larvae onto depauperate reef substratum has been shown to significantly increase larval recruitment, providing a novel option for the delivery of *ex situ* bred coral stock to the reef for restoration purposes. The success of such reef restoration approaches may be improved by the use of coral larval stock augmented for climate resilience. Here we explore whether coral climate resilience can be enhanced via interspecific hybridization through hybrid vigour. Firstly, we assessed cross-fertility of four pairs of *Acropora* species from the Great Barrier Reef. Temporal isolation in gamete release between the *Acropora* species was limited, but gametic incompatibility was present with varying strength between species pairs and depending on the direction of the hybrid crosses. We subsequently examined the fitness of hybrid and purebred larvae under heat stress by comparing their survival and settlement success throughout 10 days of exposure to 28 °C, 29.5 °C and 31 °C. Fitness of the majority of *Acropora* hybrid larvae was similar to that of the purebred larvae of both parental species, and in some instances it was higher than that of the purebred larvae of one of the parental species. Lower hybrid fertilization success did not affect larval fitness. These findings indicate that high hybrid fitness can be achieved after overcoming partial prezygotic barriers, and that interspecific hybridization may be a tool to enhance coral recruitment and climate resilience.

## Introduction

Elevated seawater temperatures, especially when above an organism’s thermal optimum, have well-documented adverse effects on marine organisms. Since 1985, coral reefs worldwide have been warming at a rate distinctly higher than the ocean average, at approximately 0.2 °C per decade^1^. Many corals live near their upper thermal tolerance limit^2^, and ocean warming is therefore detrimental to them. As for coral larvae, elevated seawater temperature is known to negatively affect their development, survival and settlement^3–5^, and larval thermal tolerance can cause a bottleneck to reef recruitment^3,6–8^. For coral recruits and adults, elevated seawater temperature can cause coral bleaching, where the symbiotic relationship between the coral host and its dinoflagellate endosymbionts (*Symbiodiniaceae*) is disrupted, often resulting in coral mortality^9^. In the last three decades, higher-than-usual seawater temperatures caused by global warming have resulted in multiple mass beaching events on coral reefs worldwide, including in 1998, 2010 and 2014–2017^1,10^. On the Great Barrier Reef (GBR), 30% coral mortality was recorded after the 2016 mass bleaching event, and a further 20% mortality was recorded following the 2017 mass bleaching event^11^. Recent estimates suggest more than 50% of the world’s coral reefs have been lost since the 1980s, and areas such as the Caribbean, Kiritimati, and certain parts of Japan have lost more than 80% of their coral^10,12^. This loss of corals directly threatens the extraordinary diversity of marine life dependent on reefs, as well as the goods and services reefs provide and that support millions of people^13,14^.

Active restoration is one possible way to restore coral cover, ecosystem function and socio-economical values of degraded coral reefs. Although current restoration attempts have not yet succeeded at a scale that can reverse global coral loss, several promising advances have been made^15–20^. For example, dela Cruz and Harrison^20^ have shown that the deployment of mature *Acropora* larvae into large scale mesh enclosures attached to the reef substratum can re-establish a breeding population of *Acropora tenuis* in three years’ time. Both larval recruitment rates and the number of surviving *Acropora* colonies two years after larval deployment were significantly higher at the reseeded sites compared to the control sites^20^. The process of coral recruitment involves the supply of larvae, the survival and settlement of these larvae, as well as post-settlement survival of the recruits^8,21^. The success of interventions such as those by Heyward *et al*.^15^ and dela Cruz and Harrison^20^ may be further improved through the use of climate resilient coral stock.

Climate resilient coral stock can potentially be produced via hybrid vigour generated from interspecific hybridization^22,23^. The benefits of hybridization have been documented extensively in commercial crops for traits of economic interest, such as yield, and disease and drought tolerance^24,25^. Hybridization creates new gene combinations and increases genetic diversity, which enhances the adaptive potential of species and their prospects of survival under environmental changes^22,26–29^, facilitates their expansion into new environments^27,30–32^ and breaks genetic correlations that constrain the evolvability of parental species^33^. For example, hybridization has led to variation in beak morphology necessary to survive environmental change in Darwin’s finches^34^, altered chemical defense systems in brassicaceae plants and assisted their survival through the Last Glacial Maximum^27^, and facilitated large scale adaptive radiation in haplochromine cichlid fishes^33^. Fitness of the first generation (F1) hybrid relative to its parental species depends on whether the gene effect is dominant (i.e., hybrid fitness is equivalent to the dominant parent, who can either be the more fit or less fit parent), additive (i.e., hybrid fitness is higher than one parent but lower than the other), over-dominant (i.e., hybrid fitness is higher than that of both parents), and under-dominant (i.e., hybrid fitness is lower than that of both parents)^25,35,36^. The scenarios where hybrids are more fit than both parents, or at least more fit than one parent are relevant for coral reef restoration.

Although the use of hybridization in conservation is limited, existing examples have demonstrated that it can rescue small, inbred populations from extinction (i.e., genetic rescue)^28,37^. These examples include the highly threatened species of Florida panther^38^, the Norfolk Island boobook owl^39^ and the Mt. Buller mountain pygmy-possum^40^. Several examples have demonstrated that interspecific hybrid corals likely represent useful stock for use in reef restoration^23,30,31^. *Acropora prolifera*, for example, the natural interspecific hybrid of *A. cervicornis* and *A. palmata* in the Caribbean, has been shown to have equivalent or higher fitness in multiple life history stages and phenotypic traits compared to the parental purebred species^41^. Similar observations were found in experimentally produced hybrids between *Acropora* species. Chan *et al*.^42^ showed that certain hybrid offspring survived better and grew faster compared to purebred offspring under ambient and elevated temperature and *p*CO_2_ conditions. Willis *et al*.^30^ reported that hybrid offspring grew faster than purebred offspring in the reef-flat environment. These examples suggest that hybrid colonies of *Acropora* are often more resilient than purebred colonies, and may represent a superior stock for reseeding of damaged reefs.

The aim of this study was to investigate whether the high hybrid fitness of *Acropora* recruits and juvenile colonies is also observed in the larval stages. To achieve this aim, we examined four experimentally crossed pairs of *Acropora* spp. from the GBR using seven parental species, and asked whether hybrid *Acropora* larvae have enhanced survival and settlement success compared to purebred larvae under ambient and elevated temperatures. As a secondary aim, we examined the extent of temporal reproductive isolation and gametic incompatibility in the four interspecific crosses of *Acropora* species.

## Results

### Spawning date and time

**Figure 1:**
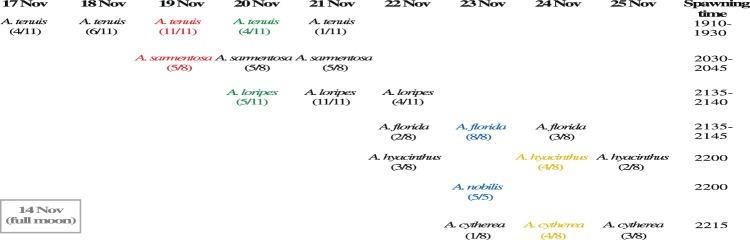
Spawning date and time of the seven *Acropora* spp. from Trunk Reef, central GBR, as observed in the SeaSim at AIMS. Brackets indicate the number of colonies spawned over the total number of colonies of that species, and colours indicate the species pairs of hybridization.

There were differences in the spawning date and time of the seven *Acropora* spp. from the central GBR that were used in this study (Fig. 1, Supplementary Table S1). *A. tenuis*, *A. loripes* and *A. sarmentosa* spawned on the earlier days after full moon (i.e., 3–8 days) and also spawned earlier in time (i.e., 19:10–21:40), whereas *A. florida*, *A. hyacinthus*, *A. nobilis* and *A. cytherea* spawned on the later days after full moon (i.e., 8–11 days) and also later in the evening (i.e., 21:35–22:15). As previously reported in the literature^23^, *A. tenuis* spawned at a distinctly earlier time (i.e., ~19:30) compared to the other species (i.e., ~20:30–22:15). Colonies of *A. loripes*, *A. florida*, *A. hyacinthus*, *A. nobilis* and *A. cytherea* all spawned within a narrow 45-minute window. Most species spawned for several consecutive days and overlapped with other species, except for *A. nobilis* where all colonies spawned on the 9^th^ day after the full moon. Most species spawned within 0.5–1.5 h since setting was observed, with the exception of *A. loripes*, which spawned between 2 and 2.5 h since setting was observed.

### Fertilization rates

**Figure 2:**
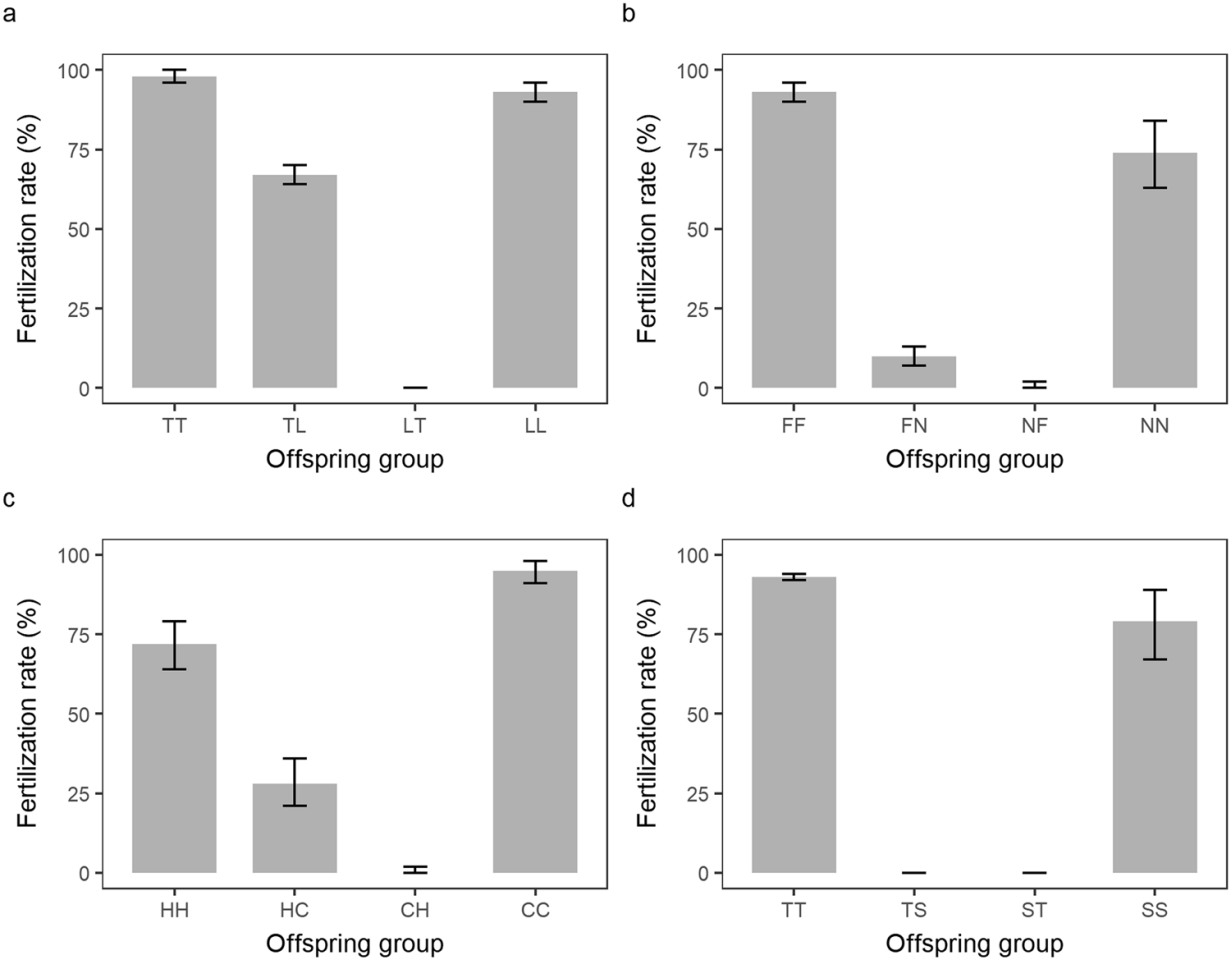
Fertilization rates for the four species pairs. (**a**) the *A. tenuis* (T) x *A. loripes* (L) cross, (**b**) the *A. florida* (F) x *A. nobilis* (N) cross, (**c**) the *A. hyacinthus* (H) x *A. cytherea* (C) cross, and (**d**) the *A. tenuis* (T) x *A. sarmentosa* cross (S). The first letter in the designation of the offspring groups represents its maternal parent species and the second letter its paternal parent species. Values are mean and error bars represent 95% CI calculated using the angular transformed data back-transformed into percentages.

**Figure 3:**
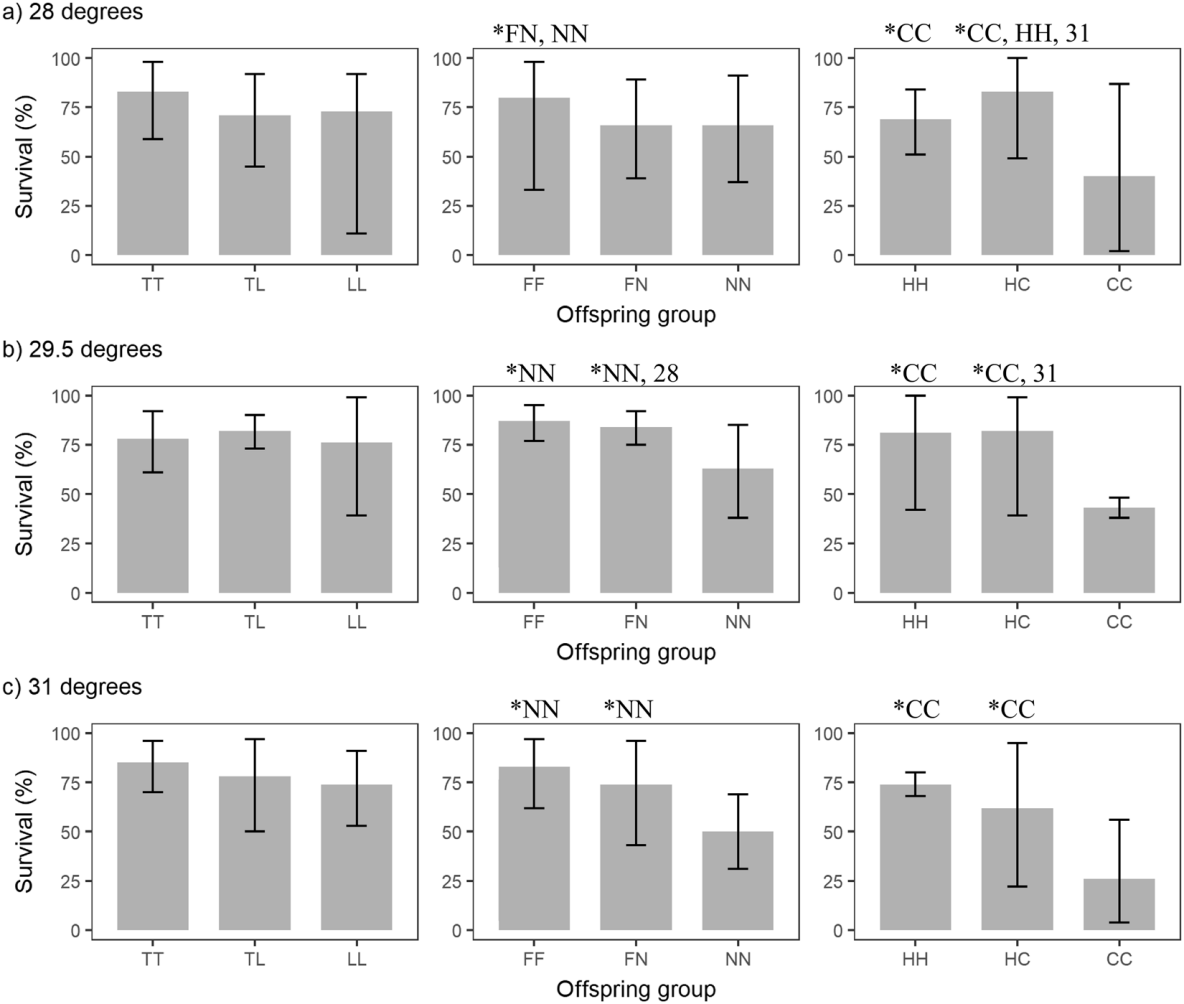
Larval survival of the offspring groups from the *A. tenuis* (T) x *A. loripes* (L) cross, the *A. florida* (F) x *A. nobilis* (N) cross, and the *A. hyacinthus* (H) x *A. cytherea* (C) cross at (**a**) 28 °C, (**b**) 29.5 °C and (**c**) 31 °C. The first letter in the designation of the offspring groups represents its maternal parent species and the second letter its paternal parent species. Values are mean and error bars represent 95% CI calculated using the angular transformed data back-transformed into percentages. ^*^Indicates significantly higher survival (i.e., p < 0.05) of this offspring group compared to the offspring group(s) indicated, or the same offspring group under the temperature treatment indicated.

Fertilization rates, measured 2.5 h after the mixing of sperm and eggs, were high for purebreds (i.e., 75–100%), and low to moderate for hybrids (i.e., 0–68%) (Fig. 2). Interspecific hybridization was successful in three out of the four *Acropora* crosses, namely (1) the *A. tenuis* x *A. loripes* cross (Fig. 2a), (2) the *A. florida* x *A. nobilis* cross (Fig. 2b), and (3) the *A. hyacinthus* x *A cytherea* cross (Fig. 2c). For these successful crosses, hybrid fertilization was only observed in one direction (i.e., eggs from parent 1 were cross-fertile with sperm from parent 2, but the reciprocal cross was unsuccessful). These included TL (65–68%), FN (9–12% and HC 24–31%) (Fig. 2). Hybrid crosses in the other direction (i.e., LT, NF and CH) showed no fertilization (i.e., 0–0.3%). For the *A. tenuis* x *A. sarmentosa* cross, hybrid crosses failed in both directions (Fig. 2d) and this cross was thus excluded from the temperature stress experiment.

### Larval survival

Survival of hybrid larvae, measured at day seven since treatment commenced, was equivalent to or higher than that of at least one parental purebred species in most cases. Out of the nine species and temperature combinations, hybrid survival was equivalent to both parents in three cases, the same as the more fit parent in four cases, higher than both parents in one case and same as the less fit parent in one case (Fig. 3, Table 1). There was no instance where hybrid survival was lower than both parents (Fig. 3, Table 1). Offspring group (i.e., the specific hybrid or purebred offspring resulting from a cross, see caption of Fig. 3) had a substantial effect on larval survival, but treatment had very limited effects. For the *A. tenuis* x *A. loripes* cross (Fig. 3, Supplementary Table S2), neither offspring group nor treatment affected larval survival. For the *A. florida* x *A. nobilis* cross (Fig. 3, Supplementary Table S3), purebred FF had higher survival than NN and hybrid FN at 28 °C (p = 0.030 for both). At 29.5 °C and 31 °C, however, survival of both the hybrid FN and purebred offspring FF was higher than that of NN (29.5 °C: p = 0.002, <0.001 respectively; 31 °C: p < 0.001 for both). For FF and NN, survival within an offspring group was not different between treatments. However, survival of hybrid FN under 29.5 °C was higher than under 28 °C (p = 0.01). For the *A. hyacinthus* x *A cytherea* cross (Fig. 3, Supplementary Table S4), survival of hybrid HC and purebred HH was higher than that of purebred CC at all three temperatures (p < 0.001 for all pairs). At 28 °C, hybrid HC also had higher survival than purebred HH (p = 0.028). For HH and CC, survival within an offspring group was unaffected by treatment. However, survival of hybrid HC at 28 °C and 29.5 °C was higher than at 31 °C (p = 0.028, 0.047 respectively). The results of an overall comparison of hybrid vs. purebred larval survival are shown in Supplementary Table S8.

**Table 1:** Summary of hybrid survival and settlement success relative to parental purebred species.

Survival of hybrids	No. of cases	Examples
Equivalent to both parents	3	TL 28 °C, TL 29.5 °C, TL 31 °C
Higher than one parent*	4	FN 29.5 °C, FN 31 °C, HC 29 °C, HC 31 °C
Higher than both parents	1	HC 28 °C
Same as the parent with the lower survival	1	FN 28 °C
Lower than both parents	0	N/A
**Settlement success of hybrids**	**No. of cases**	**Examples**
Equivalent to both parents	5	TL 28 °C, TL 29.5 °C, TL 31 °C, HC 28 °C, HC 29.5 °C
Higher than one parent^+^	3	FN 29.5 °C, FN 31 °C, HC 31 °C
Same as the parent with the lower settlement	1	FN 28 °C
Lower than both parent	0	N/A

^*^Hybrid survival was same as the more fit parent in these examples.

^+^Hybrid settlement was higher than the less fit parent but lower than the more fit parent in FN 29.5 °C, FN 31 °C.

### Larval settlement

**Figure 4:**
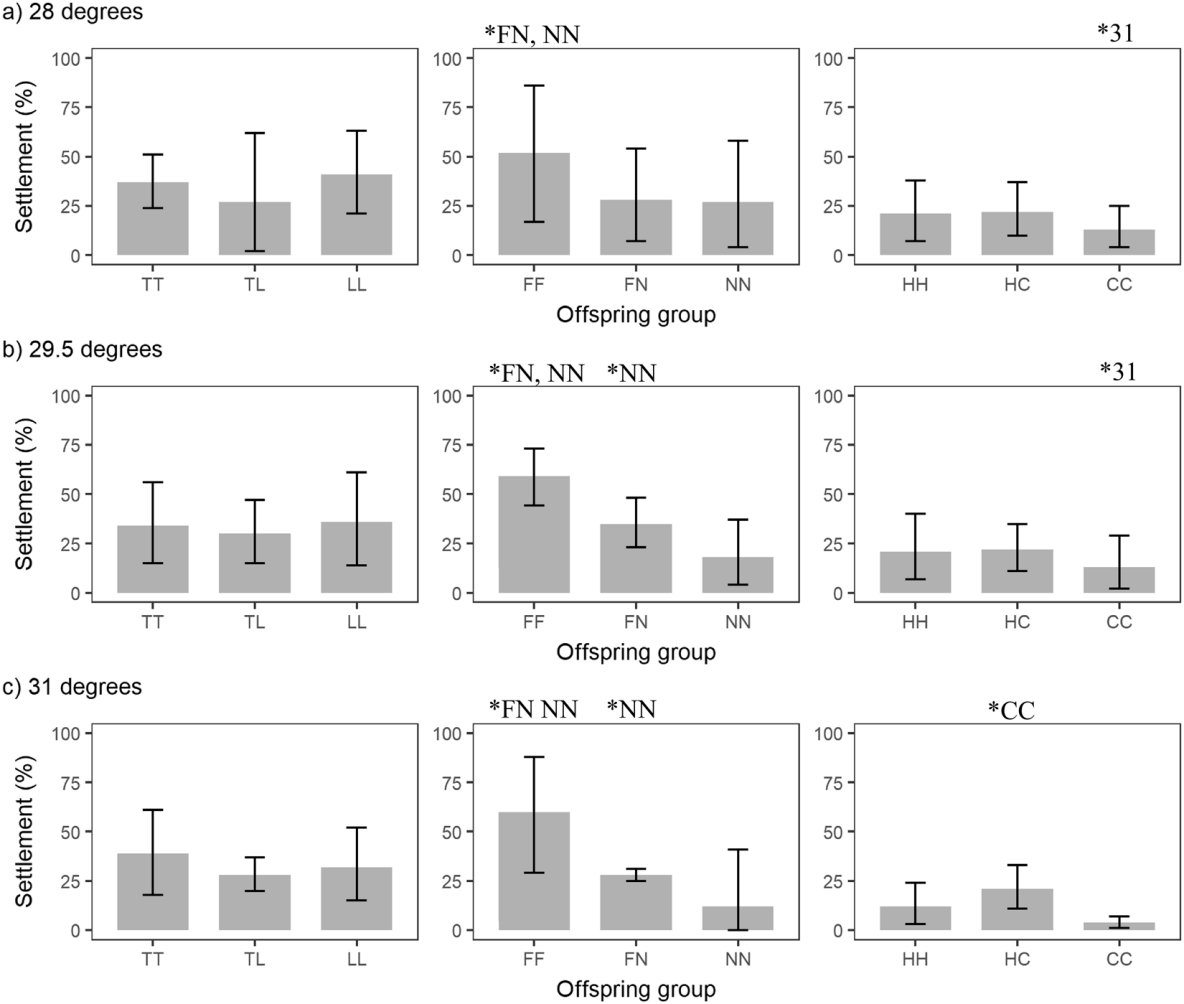
Larval settlement of the offspring groups from the *A. tenuis* (T) x *A. loripes* (L) cross, the *A. florida* (F) x *A. nobilis* (N) cross, the *A. hyacinthus* (H) x *A. cytherea* (C) cross at (**a**) 28 °C, (**b**) 29.5 °C and (**c**) 31 °C. The first letter in the designation of the offspring groups represents the origin of egg and the second letter the origin of sperm. Values are mean and error bars represent 95% CI calculated using the angular transformed data back-transformed into percentages. ^*^Indicates significantly higher survival (i.e. p < 0.05) of this offspring group compared to the offspring group (s) indicated, or the same offspring group under the temperature treatment indicated.

The larval settlement results, assessed two days after the introduction of the settlement cue, were consistent with the survival results. The majority of the hybrid larvae had settlement rates either similar to those of purebred larvae of both parental species or higher than those of purebred larvae of one parental species. Out of the nine species and temperature combinations, hybrid settlement was the same as that of purebred larvae of both parental species in five cases, more fit than purebred larvae of one parental species in three cases, and the same as that of the less fit purebred larvae of one of the parental species in one case (Fig. 4, Table 1). In the cases of the FN cross at 29.5 °C and 31 °C, settlement of hybrid FN was higher than the less fit purebred NN larvae, but lower than the more fit purebred FF larvae (i.e. additive gene effect) (Fig. 4, Table 1). In none of the cases, hybrid settlement success was lower than both parents (Fig. 4, Table 1). Offspring group (i.e., the specific hybrid or purebred offspring resulting from a cross, see caption of Fig. 4) had a substantial effect on settlement, yet treatment had very limited effect. For the *A. tenuis* x *A. loripes* cross (Fig. 4, Supplementary Table S5), larval settlement was not affected by offspring group or treatment. For the *A. florida* x *A. nobilis* cross (Fig. 4, Supplementary Table S6), the hybrid FN had a higher proportion of settled larvae than the purebred NN at 29.5 °C and 31 °C (p = 0.005, 0.008 respectively). Purebred FF also had higher settlement rates than NN, as well as FN, at all temperatures (p < 0.001 for all pairs). Treatment did not affect settlement of FN and FF, however, settlement of NN at 31 °C was significantly lower than at 28 °C (p = 0.043). For the *A. hyacinthus* x *A cytherea* cross (Fig. 4, Supplementary Table S7), the settlement rate of the hybrid HC was higher than that of the purebred CC at 31 °C (p = 0.004). For all other comparisons in this cross, settlement did not differ between offspring groups or temperatures. Abnormal settlement behavior (i.e., metamorphosis without settlement cue and without attachment to a substrate) was frequently observed in the purebred CC at 29.5 °C and 31 °C. Such behavior was not observed in the hybrid HC or the other purebred FF. The results of an overall comparison of hybrid vs. purebred larval settlement rates are shown in Supplementary Table S8.

### Seawater chemistry

Experimental conditions of the treatment are summarized in Table 2. Treatment temperatures were maintained at 28.1 °C ± 0.2, 29.5 °C ± 0.1 and 31.0 °C ± 0.2. O_2_ levels of the seawater removed from the wells ranged from 95.8 to 96.6%, indicating that the seawater remained well oxygenated throughout the experiment.

**Table 2:** Experimental conditions of the three temperature treatments measured at 12:00 daily prior to the water change. Values are mean ± SD.

Parameters*	28 °C	SD	29.5 °C	SD	31 °C	SD
Temperature (°C)	28.1	0.2	29.5	0.1	31.0	0.2
O_2_ (mg L^−1^)	7.5	0.1	7.4	0.1	7.4	0.2
O_2_(%)	95.8	1.0	96.5	0.7	96.6	2.1
pH_T_	8.14	0.01	8.12	0.02	8.12	0.01
Ω_arag_	4.47	0.08	4.56	0.11	4.72	0.16
A_T_ (µmol kg^−1^)	2378	8	2378	8	2378	8
DIC (µmol kg^−1^)	1921	8	1915	12	1903	15
Salinity (ppt)	36.5	0.2	36.5	0.2	36.6	0.2

*pH_T_ = pH in total scale; A_T_ = total alkalinity; Ω_arag_ = aragonite saturation state; DIC = dissolved inorganic carbon.

## Discussion

For sympatric broadcast spawning corals, temporal isolation and gametic incompatibility are two possible mechanisms that preclude interspecific hybridization in the wild^43,44^. Since considerable overlap in spawning date and time was observed for all but one species pairs in this study, temporal isolation is unlikely an effective prezygotic barrier. Similar observations have been reported for other *Acropora* spp.^44–46^ and *Platygyra* spp.^47^ from the GBR. However, one well-documented temporal isolation in *Acropora* spp. is that between the ‘early spawners’ and the ‘late spawners’ which are separated by about 1.5–3 h in the timing of gamete release^48–50^. The ‘early spawners’ are represented by only three species^49,50^. Relative to the 120–140 extant *Acropora* species, the existence of temporal isolation in this small group is not representative of the whole genus. Similar to the observation in Chan *et al*.^42^, the ‘early spawner’ *A. tenuis* spawned at a distinctly earlier time than all other species yet its gametes were compatible with a ‘late spawner’, *A. loripes*. Similarly, the Caribbean corals *Orbicella franksi* and *Orbicella annularis* have 2 h separation in spawning time but their gametes are compatible^51^. In both cases, a prezygotic barrier in the form of gametic incompatibility may not have evolved as the gametes are unlikely to encounter one another in nature.

Although temporal isolation was limited, gametic incompatibility was observed and its strength varied between species pairs and the direction of the hybrid cross. Fertilization rates were low to moderate in hybrid offspring groups and hybridization was only possible in one direction (i.e., asymmetric gametic incompatibility). Species-specific gametic incompatibility has previously been reported in experimental crossing of *Acropora* spp. Among 38 species pairs of *Acropora* from central GBR, eight pairs yielded high interspecific fertilization (50–80%), seven pairs had moderate fertilization (10–50%), three pairs had low fertilization (3–10%), and the remaining pairs were not cross-fertile^52,53^. Note that the fertilization rates within a species pair cross were highly variably with SDs ranging from 0 to 50%^52,53^. Experimental crosses of five *Acropora* species pairs from Okinawa (Japan) resulted in low interspecific fertilization (i.e., <2%) in all crosses, except the *A. formosa* x *A. nasuta* cross (i.e., 95%)^54^. Chan *et al*.^42^ reported high fertilization success (i.e., averaged 93%) in hybrids of both directions from *A. tenuis x A. loripes* and *A. sarmentosa* x *A. florida* crosses. Asymmetric gametic incompatibility as observed in this study is, however, not uncommon in *Acropora* spp. and has been reported in Hatta *et al*. (i.e., 40% vs. 95% in *A. formosa* x *A nasuta* cross)^54^, Fogarty *et al*. (i.e., 5–12% vs. 55–70% in *A. palmata* x *A cervicornis* cross)^44^ and Isomura *et al*. (i.e., 34% vs. 64% in *A. florida* x *A. nobilis* cross)^55^. Note that *A. intermedia* mentioned in Isomura *et al*.^55^ is the same species as *A. nobilis*. Many other taxa such as sea urchins^56^, mosquitoes^57^, tuna^58^, oak^59^ and walnut tree^60^ are also known to show asymmetric gametic incompatibility.

One possible explanation for the observed difference in gametic incompatibility is interspecific differences in gamete-recognition proteins, receptors and molecules. Gamete-recognition proteins can affect fertilization success within species^61–64^, as well as the extent of reproductive isolation between species^43,65^. Sperm proteins, such as bindin in sea urchin and sea star, and lysin in abalone, provide species-specific binding of sperm to egg and play an important role in reproductive isolation between species^43,54,66,67^. Bindin, for example, is a sperm protein in sea urchins that coats the acrosome of the sperm, binds sperm to the vitelline envelope of the egg, and facilitates the fusion of sperm and egg membranes^68,69^. Interspecific differences in bindin can result in failure of one or all of these processes, preventing fertilization from occurring^70^. In sea urchins, divergence in bindin amino acid sequence can predict gamete compatibility between species, and species with less than 1% difference in sequence are fully compatible^43^.

The complementary receptor on the egg surface (e.g., *VERL* in mollusk and *EBR1* in echinoderms) mediates species-specific sperm adhesion and also plays a role in reproductive isolation^71,72^. The receptor, however, has been much less studied due to its relatively large size compared to the sperm protein (e.g., ~4595 amino acids in the bindin receptor *EBR1* compared to 200–300 amino acids in bindin)^66^. Other than gamete-recognition proteins and receptors, species-specific diffusible molecules from the egg can also affect compatibility between species^73^. Eggs of marine invertebrates are known to produce diffusible chemo-attractants (e.g. ‘sperm-activating peptides’) that activate and attract sperm to swim toward the egg^73–76^. Abalone sperm, for example, has been shown to only respond to chemo-attractants from conspecific eggs^77^. To date, however, little is known about gamete recognition proteins and chemo-attractants in coral.

Gametic incompatibility can also vary between colonies of the same species and between locations. Hatta *et al*.^54^ and Isomura *et al*.^55^ reported interspecific fertilization rates ranged from 4–76% and 3–99% respectively between different *Acropora* colonies of the same species. Colonies of the *A. tenuis x A. loripes* cross in this study are from the same reef location as those in Chan *et al*.^42^ and crossed using similar methods. However, Chan *et al*.^42^ reported a hybridization rate of 79–95% in contrast to the 0–67% observed here. Experimental crossing of *A. florida* x *A. nobilis* yielded a fertilization rate of 34–64% in colonies from Okinawa Japan^55^, but the same cross in this study had a fertilization rate of only 0.3–10%. Further, hybridization between *M. franksi* and *M. annularis* was possible in both directions in Panama but was only possible in one direction in Bahamas^51^. We speculate that gametic incompatibilities can vary between genotypes of the same species, that minor differences in gamete-recognition proteins, receptors, and diffusible molecules associated with the gametes can exist between colonies of the same species, and that these are responsible for the variation in interspecific fertilization observed in these and our studies. For example, sperm from different individuals of the same sea urchin species has been shown to vary in chemotaxis (i.e., the ability to navigate toward the egg using chemical signals), which was demonstrated to influence individual fertilization success^78^.

Although prezygotic barriers in the form of gametic incompatibility were observed, the majority of the hybrid offspring groups were either as fit as or more fit than one of the parental purebred offspring groups, which had higher fertilization rates. This is a common phenomenon in *Acropora* species. Hybrid larvae from an *A. florida* x *A. nobilis* cross showed higher survival than purebred larvae at 5–8 days after fertilization, despite their low fertilization rate^55^. This is a critical time as *Acropora* larvae become competent for settlement and metamorphosis at about 5 days of age. High larval survival during the first week in life will thus result in a larger number of larvae that may settle. Similarly, survival of hybrid larvae and 6-week old hybrid recruits from an *A. palmata* x *A. cervicornis* cross was equivalent to that of purebreds despite lower hybrid fertilization rate^41^. The hybrids also had similar settlement rates compared to purebred larvae^41^.

We observed abnormal settlement behavior in the purebred offspring group CC under elevated temperatures, but not in the corresponding hybrid offspring group HC. Hybrid offspring that can settle normally under elevated temperatures is likely to have an advantage over some of the purebred offspring under climate change scenarios. Overall, existing evidence indicates that gametic incompatibility does not negatively affect hybrid fitness in *Acropora* corals, and the more resilient hybrid offspring may provide superior coral stock for coral reef restoration. Long term field and aquarium studies have shown higher survival and growth rate in some *Acropora* hybrids compared to purebreds^23,30^, suggesting that high hybrid fitness is not limited to the larvae but may also manifest in the later life stages.

Elevated seawater temperatures have well-documented negative effects on coral larvae in terms of larval development and motility (i.e., ciliary activity)^4^, survival^3–5^, settlement^4^, metamorphosis^69^, ability to establish symbiosis^5,79^, post-settlement mortality^80,81^, photosynthesis^3^, as well as respiration and rubisco protein expression^74^ (Table 3). Although we used treatment temperatures similar to those in the studies cited above, treatment had a limited effect on larval survival and settlement. Studies with short exposure times (i.e., 1 to 48 h) have also reported that elevated temperatures did not have a negatively impact on survival^6,81^, motility^3^, settlement, metamorphosis, photosynthesis and respiratory demand^81^, post-settlement mortality^80^, and positive effects on settlement of coral larvae was reported in some instances^80,82^ (Table 3). Most studies with longer exposure times (i.e., over 48 h), however, observed negative effects of elevated temperatures on coral larvae (Table 3). Randall and Szmant^6^ for example, showed that elevated temperatures did not affect larval survival after 48 h of exposure, but had a negative impact after 7 days of exposure. This is not the case in the present study where we used ten days of exposure time.

**Table 3:** Summary of the effects of elevated temperatures on coral larvae and recruits as reported in the literature and the present study.

Treatment	Time	Species	Larvae type	Survival	Settlement	Meta-morphosis	Res-piration	Post-settlement mortality	Reference
28 °C*, 29.5 °C, 31 °C	10 d	*A. tenuis*, *A. loripes*, *A. florida*, *A. nobiliss*, *A. hyacinthus*, *A. cytherea*	Aposymbiotic	x	x	x/-ve			This study
27 °C*, 30 °C	24 h	*Porites astreoides*	Symbiotic	x	x	x	x	−ve	^81^
25 °C+ 415 ppm*, 29 °C+ 635 ppm	9 d	*Pocillopora damicornis*	Symbiotic	x			−ve		^8^
27 °C*, 29 °C, 31 °C	3 d	*Fungia scutaria*	Symbiotic	−ve					^5^
28 °C*, 29 °C, 31 °C	48 h	*Favia fragum*	Symbiotic	x					^6^
28 °C*, 29 °C, 31 °C	7 d	*Favia fragum*	Symbiotic	−ve					^6^
20 °C, 23 °C*, 26 °C, 29 °C	5 d	*Acropora solitaryensis*	Aposymbiotic		+ve			−ve	^80^
27 °C*, 31 °C, 34 °C	1 h	*Favites chinensis*	Aposymbiotic		+ve			x	^80^
28 °C*, 30 °C, 32 °C	9 d	*Diploria strigosa*	Aposymbiotic	−ve	−ve				^4^
25 °C*, 27 °C*, 29 °C	1–11 d	*Platygyra daedalea*	Aposymbiotic		+ve				^82^
26 °C, 28 °C*, 33 °C	24 h	*Porites astreoides*	Symbiotic	−ve		−ve	−ve		^3^

^*^Represents ambient temperature in the experiment.

x: Represents no effect.

−ve: Represents a negative effect.

+ve: Represents a positive effect.

A possible explanation for the observed discrepancy may be the lower sensitivity of aposymbiotic larvae (i.e., without *Symbiodiniaceae*) to elevated temperatures compared to symbiotic larvae. The *Acropora* spp. used in the present study are broadcast spawners and their larvae are aposymbiotic. The majority of the relevant larval studies in the literature are from brooding species that release larvae already harbouring *Symbiodiniaceae* (Table 3). Symbiotic larvae are potentially more sensitive to elevated temperatures as they are exposed to reactive oxygen species (ROS) produced as by-products of photosynthesis^3,83^. Aposymbiotic larvae have been shown to have higher survival than their symbiotic counterparts of the same species under elevated temperatures^83^, possibly explaining the limited effect of temperature observed in our experiment.

Elevated temperatures may also have a delayed negative effect in later life stages that were not examined in this study. Latent negative responses to environmental stress have been documented in a variety of marine invertebrate larvae^84^. Nozawa and Harrison^80^ and Ross *et al*.^81^ showed that elevated temperatures had no or a positive effect on coral larvae initially, but were followed by high post-settlement mortality. In another coral species examined in the same experiment, however, post-settlement mortality was unaffected^80^. This hypothesis also does not hold for purebreds and hybrids of *A. tenuis* x *A. loripes* examined here, as Chan *et al*.^42^ showed that high hybrid fitness was consistently observed under seven months of exposure to elevated temperature and *p*CO_2_ conditions and no delayed negative effect was reported. Alternatively, pre-exposure to a stressor may result in preconditioning and enhance an organism’s tolerance to subsequent stress events^22,85–87^. Pre-exposure to elevated temperatures of the larvae from the present study may increase their tolerance to coral bleaching during subsequent temperature stress and possibly to a different extent in hybrid and purebred juveniles. Future longer-term studies investigating the impact of exposure of hybrid and purebred corals to sub-lethal stress on tolerance to a subsequent stress event will be invaluable.

Our findings on coral larvae show that high hybrid fitness can still be achieved after overcoming partial prezygotic barriers, and that interspecific hybridization has the potential to enhance coral recruitment and climate resilience. Although interspecific fertilization is lower than conspecific fertilization, mass-spawning corals are highly fecund and the number of larvae resulting from low or medium fertilization is still enormous. Experimental crossings of *A. palmata* and *A. cervicornis* showed low fertilization (i.e., 5–12%) in one hybrid direction^44^. Nonetheless, naturally produced hybrids of both directions are present on the reef^88^. The next important questions to investigate are whether these hybrid corals can persist in nature and continue to maintain high fitness in later generations. In the most ideal scenario, F1 hybrids are able to reproduce sexually via hybridization with other F1 hybrids and/or backcrossing with parental species. This process generates novel genotypes that are climate resilient, and high fitness may be maintained in advanced generation hybrids and backcrosses. In this case, the introduction of hybrids can bring large spatial and temporal scale benefits to the reef they are out-planted to and beyond.

Although knowledge on the reproductive potential of hybrid corals is currently limited, Isomura *et al*.^32^ have demonstrated that experimentally produced F1 hybrids of *A. intermedia* × *A. florida* were fertile and able to produce an F2 generation with high fertilization success (i.e. >80%). These hybrids were also able to backcross with either the maternal parental species only or with both parental species. Given the vast volume and great surface area of the ocean compared to laboratory conditions, the fertilization rates for F2 hybrids and backcrosses may be lower in the wild due to lower sperm concentrations^44^. Despite this, evidence of unidirectional gene flow from *A. palmata* into *A. cervicornis* in the Caribbean indicates that their hybrid *A. prolifera* is fertile and can successfully backcross with at least one parental species^88,89^.

In the case where hybrids have limited success in sexual reproduction, it is possible for hybrids to persist asexually. Fragmentation is a common way of asexual reproduction of mass spawning corals^90,91^. In the Caribbean, the hybrid *A. prolifera* is known to persist and spread across large reef areas through asexual reproduction^91^. The conservation benefits of this scenario is less than the former as the hybrids are not able to promote introgression of genes across the parental species or continue to generate novel genotypes. Nevertheless, *Acropora* corals are long-lived (up to 13–24 years for some species^92^) and F1 hybrid corals with high climate resilience may maintain ecosystem function and buy time for the reef while global warming is being addressed. In the least favorable scenario, hybrids are able to hybridize with other F1 hybrids and backcross with parental species, but hybrid breakdown (i.e., outbreeding depression) occurs in later generations. The occurrence of hybrid breakdown has been documented in certain species, although it is more commonly associated with the crossing of geographically or phenologically distant species^37,93^. If hybrid breakdown occurs, natural selection will likely remove the unfit genotypes^29,94,95^ and therefore prevent them from propagating further.

The development of novel interventions is becoming increasingly important to reef systems worldwide which are rapidly losing coral, genetic diversity and ecosystem function following multiple high mortality bleaching events. The efficacy of hybridization as a tool to produce coral stock for restoration purposes is supported by our earlier work, which demonstrated hybrid corals survived equally or better compared to purebreds and grew faster over a seven months period of exposure to ambient and elevated temperature and *p*CO_2_ conditions^23^. The next step towards safe implementation of this reef restoration intervention will be to assess F1 hybrid reproductive potential, and the fitness of F1 and advanced generation hybrids in controlled field trials.

## Materials and Methods

### Parental colony collection and *in vitro* fertilization

Parental colonies (5–11 for each species: *Acropora tenuis*, *Acropora loripes*, *Acropora florida*, *Acropora nobilis*, *Acropora hyacinthus*, *Acropora cytherea* and *Acropora sarmentosa*) were collected from Trunk Reef (18°35′S, 146°80′E), central GBR. Colonies were collected prior to the full moon on 14^th^ Nov and held in flow-through aquaria of the National Sea Simulator (SeaSim) at the Australian Institute of Marine Science (AIMS) in Townsville, Australia. When signs of imminent spawning were observed (i.e., ‘setting’, where the egg-sperm bundles of a colony are pushed to the mouth of its polyps), colonies were isolated in individual aquaria to avoid unintentional mixing of gametes prior to experimental crossing. Egg-sperm bundles from the four or five most profusely spawning colonies of a species were collected and separated using a 100 µm filter. Eggs were washed three times with filtered seawater to remove any residual sperm and placed in an individual 3 L bowl until the egg-sperm separation step was completed for all targeted colonies (within 3 h).

Similar quantities of sperm (i.e., 10^7^ sperm mL^−1^) were pooled from colonies of the same species to create a mixed sperm solution. For making the hybrid offspring, 300 mL of the pooled interspecific sperm solution was added to the eggs of each colony of the receiving species to achieve a final volume of 3 L and a sperm concentration of 10^6^ sperm mL^−1^. There were four to five replicates for each direction of the hybrid crosses, and each replicate was a different colony. Fertilization was conducted separately for each colony to avoid unintended fertilization by sperm from other conspecific colonies that was not washed away (if any). Note that self-fertilization is uncommon in *Acropora* corals. For making the purebred offspring, eggs of the conspecific colonies were pooled and 1.1 L of the pooled conspecific sperm solution was added to achieve a final volume of 11 L and a sperm concentration of 10^6^ sperm mL^−1^. There were two replicates of each purebred cross. We considered two replicates sufficient as each replicate received the same mixed eggs and sperm solution and the containers themselves were unlikely to have an effect on fertilization success. Fertilization was conducted under ambient conditions and fertilization rates were assessed at 2.5 h after introduction of the sperm.

**Figure 5:**
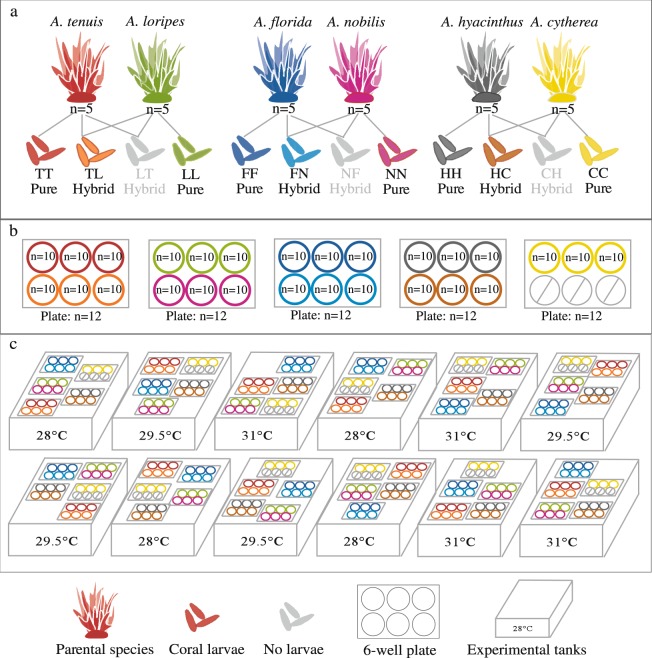
Illustrations showing the experimental setup. (**a**) The three successful *Acropora* spp. crosses (i.e., the *A. tenuis* (T) x *A. loripes* (L) cross, (2) the *A. florida* (F) x *A. nobilis* (N) cross, (3) the *A. hyacinthus* (H) *x A. cytherea* (C) cross, and the three resultant offspring groups of each cross used in the experiment, (**b**) a set of 6-well plates in each experimental tank with 3 × 10 larvae from each offspring group, and (**c**) the three temperature treatments (i.e., 28, 29.5, and 31 °C) with four replicate tanks each. The abbreviation of the offspring groups throughout this paper is that the first letter represents the origin of the eggs and the second letter the origin of sperm (e.g., TL is a hybrid crossing *A. tenuis* eggs with *A. loripes* sperm).

Four species pair crosses were carried out: (1) the *A. tenuis* x *A. loripes* cross, (2) the *A. florida* x *A. nobilis* cross, (3) the *A. hyacinthus* x *A cytherea* cross, and (4) the *A. tenuis* x *A. sarmentosa* cross. Four offspring groups were produced from each cross (i.e., two hybrid offspring groups and two purebred offspring groups, Fig. 5a). The four species pairs were selected to represent two phylogenetically divergent crosses (i.e., *A. tenuis* x *A. loripes* and *A. tenuis* x *A. sarmentosa*), and two phylogenetically closely related crosses (i.e., *A. florida* x *A. nobilis* and *A. hyacinthus* x *A cytherea*). The phylogeny of *Acropora* spp. consists of two distinct groups: the ‘early spawners’ and the ‘late spawners’, where the latter group spawns approximately 1.5–3 h earlier than the ‘early spawners’^48–50^. *A. tenuis* (early spawner) is phylogenetically divergent from *loripes* (late spawner) and *A. sarmentosa* (late spawner), while *A. florida* and *A. nobilis*, as well as *A. hyacinthus* and *A cytherea* are all late spawners and are closely related to their targeted breeding partner^48–50^. For the fertilization rate assessment, three samples of approximately 100 eggs of each offspring group taken at 2.5 h since the introduction of sperm were placed into 12-well plates and imaged using a high-resolution camera (Nikon D810). The numbers of fertilized/unfertilized embryos were visually counted. Three samples of approximately 100 eggs were also collected as self-fertilization and “no-sperm” controls in each cross conducted.

Little information is available from the literature about the relative resilience of these four parental species, but this has limited relevance for this study as our purpose was to increase genetic diversity (and thus adaptive potential) via hybridization, and not to conduct targeted breeding with species of known relative bleaching tolerance.

### Temperature stress experiment

Coral larvae were reared under ambient conditions for five days until they reached the planula stage. They remained aposymbiotic (i.e., without *Symbiodiniaceae*) throughout the experiment. The *A. tenuis* x *A. sarmentosa* interspecific cross was unsuccessful (i.e., no fertilization occurred) and thus this species pair was excluded from the experiment. For the remaining three crosses, three offspring groups (i.e., one hybrid group and two purebred groups) of each cross were used for the heat stress experiment (Fig. 5a). Hybrids in one direction of each cross were excluded due to their low fertilization success (and thus low larval yields). Using a glass pipette, planula larvae of each offspring group were carefully transferred into 6-well plates and reared under 28 °C (i.e., mean annual temperature at Davies Reef over the period 1991–2016, proximal to Trunk Reef), 29.5 °C (i.e., mean summer maximum at Davies Reef) and 31 °C (i.e., elevated temperature) (Fig. 5b). Temperatures followed the diurnal variation of 0.6 °C that typically occurs on Davies Reef and were ramped to the targeted temperatures at a rate of 0.5 °C per day. A total of 360 larvae of each of the nine offspring groups were loaded into 36 wells (i.e., 10 larvae per well) and randomly distributed among the 12 experimental tanks (Fig. 5b,c). In other words, each treatment had 120 larvae per offspring group that were distributed among 12 wells and among its four replicate tanks (Fig. 5b,c). The experimental tanks served as a water bath to maintain seawater temperatures inside the 6-well plates, and were also a seawater source for daily water change of the wells. Each treatment had four replicate tanks and five 6-well plates were placed in each tank (Fig. 5c). Positions of the tanks were randomized in the experimental room and positions of the 6-well plates were randomized within a tank (Fig. 5c).

After the larvae were transferred to the wells, the plates were covered with a lid to avoid evaporation, and floated in the treatment tanks to maintain their temperatures. Each day, 80% of the seawater of a well was exchanged using a transfer pipette. Dead or decomposing larvae that were observed during the water change were removed to maintain quality of the seawater inside the wells. Light was provided at 120 µE m^−2^ s^−1^ using Aquaillumination Hydra following the natural summer light/dark cycle.

### Larval survival and settlement

Survival and settlement of the larvae were used as proxies for fitness. Larval survival was assessed under a dissecting microscope at day seven after treatment commenced. After the survival assessment, a crustose coralline algae (CCA) chip was introduced into each well to induce larval settlement. These CCA were collected from the same reef as the parental coral colonies and maintained in flow-through aquaria at the SeaSim. On the day of larval settlement, the CCA were cut into similarly sized chips (i.e., approximately 4 mm^2^) using a bone cutter. Larval settlement rates were assessed under a dissecting microscope two days after the CCA chips were introduced. A larva was counted as ‘settled’ when it was (1) attached to a substrate (i.e., either on the surface of the well or the CCA chip) and (2) was fully metamorphosed.

### Statistical analysis

Statistical analyses were conducted separately for the *A. tenuis* x *A. loripes* cross, the *A. florida* x *A. nobilis* cross, and the *A. hyacinthus* x *A cytherea* cross using the raw data (n = 12 wells per offspring group per treatment). The response (i.e., larval survival or settlement) was treated as a binomial variable (i.e., survived/dead, settled/not settled) in the analyses. Generalized linear mixed models (GLMM)^96^ for binomial data with logistic link functions were used to test the effects of treatment and offspring group on larval survival and settlement. A random tank effect was incorporated into the models to account for possible tank effects. Models assumptions were checked visually, and models were assessed for overdispersion using a Chi-square test and goodness of fit using Akaike Information Criteria, and all of which were satisfactory. Tukey’s pairwise comparisons were then used to test for differences between treatment and offspring group and p-values of the pairwise comparisons were corrected using the Benjamini-Hochberg method. An overall comparison of hybrids vs. purebreds was also conducted using a GLMM. Statistical analyses were completed using R^97^ with packages *lme4* and *multcomp*. For illustration purpose, mean values are shown in the figures with the error bars representing 95% CI calculated using the angular transformed data that were back-transformed into percentages.

### Seawater chemistry

Automated controls of seawater temperatures were provided by SeaSim via the SCADA (Supervisory Control and Data Acquisition) system. Seawater temperature of each tank was monitored hourly using resistance temperature detector (RTD). To confirm the treatment conditions inside the 6-well plates (i.e., where the larvae were located), seawater that was removed from the wells during water change was collected for measurement of O_2_ level, salinity, temperature and pH every day at 12:00 using the HACH HQ40D Portable Multi Meter. Salinity measurements were calibrated with IAPSO Standard Seawater. Seawater from several wells of the same tank was combined for measurement due to depth requirement of the measurement probes. Total alkalinity (A_T_) was measured twice during the 10-day experiment using VINDTA calibrated to Dickson’s Certified Reference Material. Ω_arag_ (aragonite saturation state) and DIC (dissolved inorganic carbon) were calculated using the measured values of seawater A_T_, pH, temperature and salinity, with the program CO2SYS^98^ as implemented in Microsoft Excel by Pierrot *et al*.^99^.

## Supplementary information

Supplementary information

## Data Availability

The datasets generated during the present study are publicly available via the Australian Institute of Marine Science data at: https://apps.aims.gov.au/metadata/view/69f17afe-378b-41a2-8c90-5fff3318898c.
