# Supplementary material for: Interspecific gamete compatibility and hybrid larval fitness in reef-building corals: Implications for coral reef restoration
Source: Sci Rep. 2019 Mar 18;9:4757. doi: 10.1038/s41598-019-41190-5 (PMC6426996; doi:10.1038/s41598-019-41190-5)
Supplement: Supplementary file 1 — Supplementary information [file 41598_2019_41190_MOESM1_ESM.docx]

**Interspecific gamete compatibility and hybrid larval fitness in reef-building corals: Implications for coral reef restoration**

**Supplementary information**

**Supplementary Table S1.** Spawning date and time of the seven *Acropora* spp. from Trunk Reef, central GBR.

| **Date** | **Species** | **Days after full moon** | **No. of colonies** | **Setting time** | **Spawning time** |
| --- | --- | --- | --- | --- | --- |
| 17/11/2016 | *A. tenuis* | 3 | 4/11 | 18:30 | 19:30 |
| 18/11/2016 | *A. tenuis* | 4 | 6/11 | 18:30 | 19:30 |
| 19/11/2016 | *A. tenuis* | 5 | 11/11 | 18:30 | 19:30 |
| 20/11/2016 | *A. tenuis* | 6 | 4/11 | 18:15 | 19:10-19:30 |
| 21/11/2016 | *A. tenuis* | 7 | 1/11 | 18:00 | 19:30 |
|  |  |  |  |  |  |
| 18/11/2016 | *A. loripes* | 4 | 1/11 | 20:20 | No spawning |
| 19/11/2016 | *A. loripes* | 5 | 2/11 | 20:20 | No spawning |
| 20/11/2016 | *A. loripes* | 6 | 5/11 | 19:30 | 21:35 |
| 21/11/2016 | *A. loripes* | 7 | 11/11 | 19:15 | 21:40 |
| 22/11/2016 | *A. loripes* | 8 | 4/11 | 19:30 | 21:40 |
|  |  |  |  |  |  |
| 19/11/2016 | *A. sarmentosa* | 5 | 5/8 | 19:30 | 20:30 |
| 20/11/2016 | *A. sarmentosa* | 6 | 5/8 | 19:00 | 20:45 |
| 21/11/2016 | *A. sarmentosa* | 7 | 5/8 | 19:00 | 20:30 |
|  |  |  |  |  |  |
| 19/11/2016 | *A. florida* | 5 | 2/8 | 21:00 | No spawning |
| 20/11/2016 | *A. florida* | 6 | 2/8 | 19:50 | No spawning |
| 21/11/2016 | *A. florida* | 7 | 3/8 | 21:30 | No spawning |
| 22/11/2016 | *A. florida* | 8 | 2/8 | 21:00 | 21:35 |
| 23/11/2016 | *A. florida* | 9 | 8/8 | 19:50 | 21:45 |
| 24/11/2016 | *A. florida* | 10 | 3/8 | 21:00 | 21:45 |
|  |  |  |  |  |  |
| 22/11/2016 | *A. hyacinthus* | 8 | 3/8 | 19:50 | 22:00 |
| 24/11/2016 | *A. hyacinthus* | 10 | 4/8 | 20:00-21:00 | 22:00 |
| 25/11/2016 | *A. hyacinthus* | 11 | 2/8 | 20:30 | 22:00 |
|  |  |  |  |  |  |
| 23/11/2016 | *A. nobilis* | 9 | 5/5 | 20:00-20:30 | 22:00 |
|  |  |  |  |  |  |
| 23/11/2016 | *A. cytherea* | 9 | 1/8 | No setting | 22:15 |
| 24/11/2016 | *A. cytherea* | 10 | 4/8 | 20:30-21:30 | 22:15 |
| 25/11/2016 | *A. cytherea* | 11 | 3/8 | 20:45 | 22:15 |

**Supplementary Table S2.** Tukey’s pairwise comparisons on the effect of offspring group (i.e., TT, TL and LL) and treatment (i.e., 28 ºC, 29 ºC and 31 ºC) on larval survival of the *A. tenuis* (T) x *A. loripes* (L) cross. The first letter in the abbreviation of the offspring groups represents the origin of the egg and the second letter the origin of sperm. An odds ratio of > 1 indicates higher survival, and < 1 indicates lower survival of the first offspring group/ treatment in comparison.

| **Comparison** | | | **Log odds ratio** | **Std. error** | **z-value** | **p-value** | **Odds ratio** |
| --- | --- | --- | --- | --- | --- | --- | --- |
| TL.28 | - | TT.28 | -0.76 | 0.32 | -2.35 | 0.644 | 0.47 |
| LL.28 | - | TT.28 | -0.63 | 0.33 | -1.92 | 0.655 | 0.53 |
| LL.28 | - | TL.28 | 0.13 | 0.30 | 0.45 | 0.842 | 1.14 |
| TL.29.5 | - | TT.29.5 | 0.21 | 0.33 | 0.66 | 0.842 | 1.24 |
| LL.29.5 | - | TT.29.5 | -0.15 | 0.31 | -0.47 | 0.842 | 0.86 |
| LL.29.5 | - | TL.29.5 | -0.36 | 0.32 | -1.12 | 0.842 | 0.70 |
| TL.31 | - | TT.31 | -0.51 | 0.34 | -1.51 | 0.792 | 0.60 |
| LL.31 | - | TT.31 | -0.70 | 0.33 | -2.10 | 0.644 | 0.50 |
| LL.31 | - | TL.31 | -0.19 | 0.31 | -0.62 | 0.842 | 0.83 |
|  |  |  |  |  |  |  |  |
| TT.29.5 | - | TT.28 | -0.39 | 0.48 | -0.81 | 0.842 | 0.68 |
| TT.31 | - | TT.28 | 0.09 | 0.50 | 0.18 | 0.915 | 1.09 |
| TT.31 | - | TT.29.5 | 0.48 | 0.48 | 0.99 | 0.842 | 1.61 |
| TL.29.5 | - | TL.28 | 0.59 | 0.46 | 1.27 | 0.818 | 1.80 |
| TL.31 | - | TL.28 | 0.34 | 0.46 | 0.75 | 0.842 | 1.40 |
| TL.31 | - | TL.29.5 | -0.25 | 0.47 | -0.53 | 0.842 | 0.78 |
| LL.29.5 | - | LL.28 | 0.10 | 0.46 | 0.21 | 0.915 | 1.10 |
| LL.31 | - | LL.28 | 0.02 | 0.45 | 0.04 | 0.967 | 1.02 |
| LL.31 | - | LL.29.5 | -0.08 | 0.45 | -0.17 | 0.915 | 0.93 |

* indicates significant difference in this comparison.

**Supplementary Table S3.** Tukey’s pairwise comparisons on the effect of offspring group (i.e., FF, FN and NN) and treatment (i.e., 28 ºC, 29 ºC and 31 ºC) on larval survival of the *A. florida* (F) x *A. nobilis* (N) cross. The first letter in the abbreviation of the offspring groups represents the maternal parent species and the second letter the paternal parent species. An odds ratio of > 1 indicates higher survival, and < 1 indicates lower survival of the first offspring group/ treatment in comparison.

| **Comparison** | | | **Log odds ratio** | **Std. error** | **z-value** | **p-value** | **Odds ratio** |
| --- | --- | --- | --- | --- | --- | --- | --- |
| FN.28 | - | FF.28* | -0.73 | 0.30 | -2.46 | 0.030 | 0.48 |
| NN.28 | - | FF.28* | -0.73 | 0.30 | -2.46 | 0.030 | 0.48 |
| NN.28 | - | FN.28 | 0.00 | 0.27 | 0.00 | 1.000 | 1.00 |
| FN.29.5 | - | FF.29.5 | -0.20 | 0.37 | -0.55 | 0.676 | 0.82 |
| NN.29.5 | - | FF.29.5* | -1.34 | 0.33 | -4.05 | < 0.001 | 0.26 |
| NN.29.5 | - | FN.29.5* | -1.13 | 0.31 | -3.60 | 0.002 | 0.32 |
| FN.31 | - | FF.31 | -0.50 | 0.32 | -1.57 | 0.175 | 0.61 |
| NN.31 | - | FF.31* | -1.57 | 0.30 | -5.17 | < 0.001 | 0.21 |
| NN.31 | - | FN.31* | -1.07 | 0.28 | -3.83 | < 0.001 | 0.34 |
|  |  |  |  |  |  |  |  |
| FF.29.5 | - | FF.28 | 0.49 | 0.39 | 1.28 | 0.273 | 1.64 |
| FF.31 | - | FF.28 | 0.18 | 0.37 | 0.49 | 0.704 | 1.20 |
| FF.31 | - | FF.29.5 | -0.31 | 0.39 | -0.80 | 0.523 | 0.73 |
| FN.29.5 | - | FN.28* | 1.03 | 0.35 | 2.91 | 0.011 | 2.79 |
| FN.31 | - | FN.28 | 0.41 | 0.32 | 1.27 | 0.273 | 1.51 |
| FN.31 | - | FN.29.5 | -0.61 | 0.36 | -1.70 | 0.147 | 0.54 |
| NN.29.5 | - | NN.28 | -0.11 | 0.31 | -0.35 | 0.772 | 0.90 |
| NN.31 | - | NN.28 | -0.66 | 0.31 | -2.14 | 0.058 | 0.52 |
| NN.31 | - | NN.29.5 | -0.55 | 0.31 | -1.80 | 0.123 | 0.58 |

* indicates significant difference in this comparison.

**Supplementary Table S4.** Tukey’s pairwise comparisons on the effect of offspring group (i.e., HH, HC and CC) and treatment (i.e., 28 ºC, 29 ºC and 31 ºC) on larval survival of the *A. hyacinthus* (H) x *A. cytherea* (C) cross. The first letter in the abbreviation of the offspring groups represents the origin of the egg and the second letter the origin of sperm. An odds ratio of > 1 indicates higher survival, and < 1 indicates lower survival of the first offspring group/ treatment in comparison.

| **Comparison** | | | **Log odds ratio** | **Std. error** | **z-value** | **p-value** | **Odds ratio** |
| --- | --- | --- | --- | --- | --- | --- | --- |
| HC.28 | - | HH.28* | 0.79 | 0.32 | 2.46 | 0.028 | 2.20 |
| CC.28 | - | HH.28* | -1.31 | 0.29 | -4.61 | < 0.001 | 0.27 |
| CC.28 | - | HC.28* | -2.10 | 0.32 | -6.60 | < 0.001 | 0.12 |
| HC.29.5 | - | HH.29.5 | 0.06 | 0.33 | 0.17 | 0.867 | 1.06 |
| CC.29.5 | - | HH.29.5* | -1.76 | 0.30 | -5.85 | < 0.001 | 0.17 |
| CC.29.5 | - | HC.29.5* | -1.82 | 0.30 | -5.97 | < 0.001 | 0.16 |
| CC.31 | - | HH.31* | -2.21 | 0.30 | -7.26 | < 0.001 | 0.11 |
| CC.31 | - | HC.31* | -1.61 | 0.29 | -5.55 | < 0.001 | 0.20 |
| HC.31 | - | HH.31 | -0.60 | 0.29 | -2.11 | 0.058 | 0.55 |
|  |  |  |  |  |  |  |  |
| HH.29.5 | - | HH.28 | 0.61 | 0.47 | 1.31 | 0.253 | 1.84 |
| HH.31 | - | HH.28 | 0.23 | 0.46 | 0.51 | 0.686 | 1.26 |
| HH.31 | - | HH.29.5 | -0.38 | 0.47 | -0.81 | 0.487 | 0.68 |
| HC.29.5 | - | HC.28 | -0.12 | 0.49 | -0.25 | 0.828 | 0.89 |
| HC.31 | - | HC.28* | -1.16 | 0.47 | -2.48 | 0.028 | 0.31 |
| HC.31 | - | HC.29.5* | -1.04 | 0.46 | -2.25 | 0.047 | 0.35 |
| CC.29.5 | - | CC.28 | 0.16 | 0.44 | 0.37 | 0.759 | 1.18 |
| CC.31 | - | CC.28 | -0.66 | 0.45 | -1.47 | 0.203 | 0.52 |
| CC.31 | - | CC.29.5 | -0.83 | 0.45 | -1.85 | 0.101 | 0.44 |

* indicates significant difference in this comparison.

**Supplementary Table S5.** Tukey’s pairwise comparisons on the effect of offspring group (i.e., TT, TL and LL) and treatment (i.e., 28 ºC, 29 ºC and 31 ºC) on larval settlement of the *Acropora tenuis* (T) x *Acropora loripes* (L) cross. The first letter in the abbreviation of the offspring groups represents the origin of the egg and the second letter the origin of sperm. An odds ratio of > 1 indicates higher survival, and < 1 indicates lower survival of the first offspring group/ treatment in comparison.

| **Comparison** | | | **Log odds ratio** | **Std. error** | **z-value** | **p-value** | **Odds ratio** |
| --- | --- | --- | --- | --- | --- | --- | --- |
| TL.28 | - | TT.28 | -0.50 | 0.28 | -1.79 | 0.481 | 0.61 |
| LL.28 | - | TT.28 | 0.14 | 0.26 | 0.53 | 0.767 | 1.15 |
| LL.28 | - | TL.28 | 0.64 | 0.28 | 2.31 | 0.481 | 1.90 |
| TL.29.5 | - | TT.29.5 | -0.19 | 0.28 | -0.69 | 0.767 | 0.83 |
| LL.29.5 | - | TT.29.5 | 0.07 | 0.27 | 0.27 | 0.792 | 1.08 |
| LL.29.5 | - | TL.29.5 | 0.26 | 0.28 | 0.96 | 0.685 | 1.30 |
| TL.31 | - | TT.31 | -0.49 | 0.28 | -1.77 | 0.481 | 0.61 |
| LL.31 | - | TT.31 | -0.33 | 0.27 | -1.21 | 0.579 | 0.72 |
| LL.31 | - | TL.31 | 0.16 | 0.28 | 0.56 | 0.767 | 1.17 |
|  |  |  |  |  |  |  |  |
| TT.29.5 | - | TT.28 | -0.15 | 0.27 | -0.54 | 0.767 | 0.86 |
| TT.31 | - | TT.28 | 0.07 | 0.27 | 0.27 | 0.792 | 1.07 |
| TT.31 | - | TT.29.5 | 0.22 | 0.27 | 0.80 | 0.730 | 1.24 |
| TL.29.5 | - | TL.28 | 0.16 | 0.29 | 0.57 | 0.767 | 1.18 |
| TL.31 | - | TL.28 | 0.08 | 0.29 | 0.29 | 0.792 | 1.09 |
| TL.31 | - | TL.29.5 | -0.08 | 0.28 | -0.28 | 0.792 | 0.92 |
| LL.29.5 | - | LL.28 | -0.21 | 0.27 | -0.80 | 0.730 | 0.81 |
| LL.31 | - | LL.28 | -0.40 | 0.27 | -1.47 | 0.506 | 0.67 |
| LL.31 | - | LL.29.5 | -0.19 | 0.27 | -0.68 | 0.767 | 0.83 |

* indicates significant difference in this comparison.

**Supplementary Table S6.** Tukey’s pairwise comparisons on the effect of offspring group (i.e., FF, FN and NN) and treatment (i.e., 28 ºC, 29 ºC and 31 ºC) on larval settlement of the *A. florida* (F) x *A. nobilis* (N) cross. The first letter in the abbreviation of the offspring groups represents their maternal parent species and the second letter their paternal parent species. An odds ratio of > 1 indicates higher survival, and < 1 indicates lower survival of the first offspring group/ treatment in comparison.

| **Comparison** | | | **Log odds ratio** | **Std. error** | **z-value** | **p-value** | **Odds ratio** |
| --- | --- | --- | --- | --- | --- | --- | --- |
| FN.28 | - | FF.28* | -1.04 | 0.28 | -3.71 | < 0.001 | 0.35 |
| NN.28 | - | FF.28* | -1.12 | 0.28 | -3.98 | < 0.001 | 0.33 |
| NN.28 | - | FN.28 | -0.09 | 0.29 | -0.29 | 0.838 | 0.92 |
| FN.29.5 | - | FF.29.5* | -1.00 | 0.27 | -3.73 | < 0.001 | 0.37 |
| NN.29.5 | - | FF.29.5* | -1.94 | 0.30 | -6.36 | < 0.001 | 0.14 |
| NN.29.5 | - | FN.29.5* | -0.94 | 0.31 | -3.05 | 0.005 | 0.39 |
| FN.31 | - | FF.31* | -1.40 | 0.28 | -5.01 | < 0.001 | 0.25 |
| NN.31 | - | FF.31* | -2.39 | 0.34 | -7.12 | < 0.001 | 0.09 |
| NN.31 | - | FN.31* | -0.99 | 0.35 | -2.87 | 0.008 | 0.37 |
|  |  |  |  |  |  |  |  |
| FF.29.5 | - | FF.28 | 0.30 | 0.35 | 0.88 | 0.429 | 1.36 |
| FF.31 | - | FF.28 | 0.34 | 0.35 | 0.99 | 0.401 | 1.41 |
| FF.31 | - | FF.29.5 | 0.04 | 0.35 | 0.11 | 0.935 | 1.04 |
| FN.29.5 | - | FN.28 | 0.34 | 0.36 | 0.94 | 0.401 | 1.41 |
| FN.31 | - | FN.28 | -0.02 | 0.37 | -0.06 | 0.953 | 0.98 |
| FN.31 | - | FN.29.5 | -0.36 | 0.36 | -1.01 | 0.401 | 0.70 |
| NN.29.5 | - | NN.28 | -0.51 | 0.39 | -1.30 | 0.268 | 0.60 |
| NN.31 | - | NN.28* | -0.93 | 0.42 | -2.23 | 0.043 | 0.40 |
| NN.31 | - | NN.29.5 | -0.41 | 0.43 | -0.96 | 0.401 | 0.66 |

* indicates significant difference in this comparison.

**Supplementary Table S7.** Tukey’s pairwise comparisons on the effect of offspring group (i.e., HH, HC and CC) and treatment (i.e., 28 ºC, 29 ºC and 31 ºC) on larval settlement of the *A. hyacinthus* (H) x *A. cytherea* (C) cross. The first letter in the abbreviation of the offspring groups represents the origin of the egg and the second letter the origin of sperm. An odds ratio of > 1 indicates higher survival, and < 1 indicates lower survival of the first offspring group/ treatment in comparison.

| **Comparison** | | | **Log odds ratio** | **Std. error** | **z-value** | **p-value** | **Odds ratio** |
| --- | --- | --- | --- | --- | --- | --- | --- |
| HC.28 | - | HH.28 | 0.05 | 0.32 | 0.16 | 0.984 | 1.05 |
| CC.28 | - | HH.28 | -0.61 | 0.36 | -1.72 | 0.166 | 0.54 |
| CC.28 | - | HC.28 | -0.66 | 0.35 | -1.87 | 0.149 | 0.52 |
| HC.29.5 | - | HH.29.5 | 0.05 | 0.32 | 0.16 | 0.984 | 1.05 |
| CC.29.5 | - | HH.29.5 | -0.54 | 0.35 | -1.53 | 0.196 | 0.58 |
| CC.29.5 | - | HC.29.5 | -0.59 | 0.35 | -1.69 | 0.166 | 0.56 |
| HC.31 | - | HH.31 | 0.69 | 0.36 | 1.90 | 0.149 | 1.99 |
| CC.31 | - | HH.31 | -1.09 | 0.54 | -2.02 | 0.149 | 0.34 |
| CC.31 | - | HC.31* | -1.77 | 0.51 | -3.48 | 0.004 | 0.17 |
|  |  |  |  |  |  |  |  |
| HH.29.5 | - | HH.28 | 0.00 | 0.32 | 0.00 | 1.000 | 1.00 |
| HH.31 | - | HH.28 | -0.69 | 0.36 | -1.90 | 0.149 | 0.50 |
| HH.31 | - | HH.29.5 | -0.69 | 0.36 | -1.90 | 0.149 | 0.50 |
| HC.29.5 | - | HC.28 | 0.00 | 0.31 | 0.00 | 1.000 | 1.00 |
| HC.31 | - | HC.28 | -0.05 | 0.32 | -0.16 | 0.984 | 0.95 |
| HC.31 | - | HC.29.5 | -0.05 | 0.32 | -0.16 | 0.984 | 0.95 |
| CC.29.5 | - | CC.28 | 0.07 | 0.39 | 0.19 | 0.984 | 1.08 |
| CC.31 | - | CC.28 | -1.16 | 0.53 | -2.18 | 0.149 | 0.31 |
| CC.31 | - | CC.29.5 | -1.24 | 0.53 | -2.33 | 0.118 | 0.29 |

* indicates significant difference in this comparison.

**Supplementary Table S8.** Overall comparisons of hybrid vs. purebred larval survival and settlement across temperatures. An odds ratio of > 1 indicates higher survival of purebreds, and < 1 indicates lower survival of purebreds compared to hybrids. Due to the extreme low survival and settlement of offspring group CC, the analyses were tested with and without incorporating offspring group CC.

| **Trait** | **Comparison** | | | **Log odds ratio** | **Std. error** | **z-value** | **p-value** | **Odds ratio** |
| --- | --- | --- | --- | --- | --- | --- | --- | --- |
| Survival | Purebreds | - | Hybrids* | -0.36 | 0.09 | -4.25 | <0.001 | 0.70 |
| Survival# | Purebreds | - | Hybrids | -0.04 | 0.09 | -0.44 | 0.661 | 0.93 |
| Settlement | Purebreds | - | Hybrids | 0.14 | 0.08 | 1.68 | 0.094 | 1.15 |
| Settlement# | Purebreds | - | Hybrids* | 0.32 | 0.09 | 3.75 | <0.001 | 1.38 |

* indicates significant difference in this comparison.

# Offspring group CC was removed from this analysis.
